# “Data is the new oil”: citizen science and informed consent in an era of researchers handling of an economically valuable resource

**DOI:** 10.1186/s40504-021-00118-6

**Published:** 2021-12-10

**Authors:** Etain Quigley, Ingrid Holme, David M. Doyle, Aileen K. Ho, Eamonn Ambrose, Katie Kirkwood, Gerardine Doyle

**Affiliations:** 1 Department of Law, Maynooth University, Kildare, Ireland; 2 School of Sociology, University College Dublin, Dublin, Ireland; 3 Department of Law, Maynooth University, maynooth, Kildare Ireland; 4 School of Psychology and Clinical Language Sciences, Reading University, Earley, Reading, RG6 7BE UK; 5 School of Business, University College Dublin, Belfield, Dublin Ireland; 6 Department of Law, Maynooth University, Kildare, Ireland; 7 Michael Smurfit Graduate Business School, University College Dublin, Dublin Ireland

**Keywords:** Huntington’s disease, Data, GDPR, Ethics, Citizen science

## Abstract

As with other areas of the social world, academic research in the contemporary healthcare setting has undergone adaptation and change. For example, research methods are increasingly incorporating citizen participation in the research process, and there has been an increase in collaborative research that brings academic and industry partners together. There have been numerous positive outcomes associated with both of these growing methodological and collaborative processes; nonetheless, both bring with them ethical considerations that require careful thought and attention. This paper addresses the ethical considerations that research teams must consider when using participatory methods and/or when working with industry and outlines a novel informed consent matrix designed to maintain the high ethical standard to which academic research in the healthcare arena has traditionally adhered.

## Introduction

The ethical dilemmas that arise from a researchers’ perspective, and dilemmas that must be considered by researchers, are numerous. This is particularly so in the healthcare setting and in an era of technology where academic/industry collaboration is becoming the norm. A typical academic research project runs by gaining explicit informed consent at the outset of the project to collect specific personal data that will be used for a specific project. The academic researchers control the data within the academic space; they analyse the data and produce findings. However, in the technological age, and as a result of the growing push for academic and non-academic research collaboration, these steps have altered, and researchers are responsible for ensuring that these alterations do not impact the data subject and their privacy rights. This paper outlines the process of developing a fit for purpose informed consent protocol for a citizen science project that expands beyond the traditional academic boundaries by incorporating additional considerations when working with industry partners.

### Citizen science

There has been an increasing shift towards the inclusion of study participants as more than passive research participants by recognising them as key stakeholders and valuable research partners (Woolley et al. 2016). This is no more evident than in the citizen science model of research. The European Commission has defined the citizen science model as gathering data by non-professionals and non-experts as part of research studies and scientific experimentation (Palazzani et al. 2015). Others have outlined citizen science as ‘the active participation of lay people in scientific research’ and ‘the participation of non-professionals at any phase of scientific research’ (Broeder et al. 2017; Fiske et al. 2019). This has been described as being ‘widely celebrated for producing creative synergies of lay and expert collaboration, or even making science more democratic’ (Fiske et al. 2019). Citizen science has increasingly gained traction, especially since the 1995 publication of *Citizen Science* by Alan Irwin and can be broken into three strands: 1) involving lay people in biology, conservation and ecology; 2) engaging citizens in the collection of geographical data, and 3) social science and epidemiology (Suman and Pierce 2018). It is this third strand that forms the basis of this paper.

Participant inclusion in research projects is not new. A more traditional approach to participant inclusion is self-reporting data through the likes of surveys; yet, the data subject only plays a limited role in this type of approach and is a passive actor in the project overall. Under the citizen science model, the citizen becomes a key stakeholder in terms of research design, generating data, analysing data and validating data alongside the research team. Such approaches have been used to bring typically distrustful cohorts into the research space, thus broadening the participant pool which in turn leads to greater representativeness in research (Skinner et al. 2015). Within the healthcare setting, this collaborative process is no more evident than in the area of Connected Health (healthcare technology). For example, collecting personal data through smart devices which are part of a study participant’s daily life, makes the participant part of the data gathering team and indeed their role in the analysis can often be greater than in non-Connected Health research as the participant has to describe the data and put it into context for the researcher. Finally, it has become a typical practice to return to the data subject to seek validation on the data collected. This method of collecting, analysing and validating data has the potential to expose the problems with any Connected Health intervention from the user’s perspective. It thus provides an opportunity for the research team to identify possible solutions and incorporate feedback on an ongoing basis (Rowbotham et al. 2019). Moreover, this form of collaboration gives the data subjects a new active role in the process whereby they are no longer passive agents.

### Citizen science and informed consent

What has been discussed highlights changes to the research process and with that change brings new considerations, particularly around ethics. Concerns in this area are not new with scholars raising issues such as balancing the community with the individual; power relationships and dynamics; working with vulnerable and/or stigmatized persons; working through conflicting ethical issues; and issues around social action resulting from research outputs (Kwan and Walsh 2018). Thus, the citizen science model includes additional ethical considerations, particularly around preventing harm to participants and obtaining consent at all stages of the research project and data usage. Under Article 4 of the General Data Protection Regulation (GDPR), consent is not valid unless it is freely given, specific to the use for which it was collected, informed, unambiguous and easily withdrawn. This is particularly important if the project involves special categories of personal data (e.g. health data), and therefore requires an additional degree of protection from the data processors in light of the requirements imposed by Article 9 of the GDPR. Without informed consent, the ethical standing of the project fails, and the project moves into the realm of covert data collection (Niekerk and Albert 2014). Having informed consent facilitates the collection, use and processing of personal data in a manner that respects the study participants’ ownership of this data and allows them to retain control over their data even after it has been provided to the study (Harlow et al. 2020). Informed consent is a process whereby the individual is fully informed of the nature and specific purpose of the project, the data that is being collected, and how that data will be used subsequent to their participation. It is based on the doctrine of ‘individual autonomy, dignity, and integrity, rooted in the fundamental respect for a person, and intertwined with the right to respect privacy’ (Cheung 2018). However, informed consent is not a simple panacea that protects against paternalism and autocratic practices whereby the manner in which informed consent is obtained and utilised during a project can vary from vague and limiting to engaging and empowering (Corrigan 2003). These concepts are particularly important with regard to citizen science and healthcare research because of the type of data being collected (sensitive) and the commercially valuable nature of the data (Palazzani et al. 2015).

When conducting research in the healthcare field, informed consent becomes increasingly important for many reasons but three primary concerns arise: first, when you are dealing with data, you are working with information pertaining to human beings, and every one of those research participants is recognised as having inherent rights that should be protected; secondly, the data collected can be of a very sensitive nature and can have serious implications for the giver of the data if used outside of the parameters to which they consented; thirdly, this data is often commercially valuable, and as such, researchers are in a sense holding economically valuable and commercially powerful information. The latter becomes even more relevant when researchers of non-commercial institutions, such as universities, collaborate with commercial institutions, such as industry, or where data is transferred out of the E.U.

In a typical study, informed consent is sought by the researcher following a sequence of information providing sessions. The assumption underpinning informed consent is that the data subject’s rights and welfare are protected by providing a space for the data subject to make free and informed decisions regarding themselves and this must form a central pillar of all informed consent processes (Corrigan 2003). Further, informed consent usually relates to one study which is conducted at an institution for a specific purpose (Kuehn 2013). In the age of platformed technologies, research has become more complex, not least because the data is no longer controlled solely by the researcher, and this creates additional challenges and ethical considerations. For example, technological advances have led to the collection of big data, to previously unthinkable data analytics, and the use of data mining, all leading to health and medical research becoming ‘data-intensive, global, and virtual’ (Cheung 2018; Schmietow 2016). Furthermore, there are additional concerns, as the control of data has changed, and the researcher’s role in terms of data controller has altered. For example, when technology forms part of the research project, the unforeseen use of the data is often outside of the control of the original researcher (Schmietow 2016), leading some to suggest that truly informed consent is now incompatible with big data research (Froomkin 2019). In addition, the risks attached to subsequent use of data may not be known during the original research informed consent processes (Schmietow 2016). As a result, the informed consent process appears to be somewhat diluted, and the ethical position of the researcher may be at risk.

It should also be borne in mind that all of the data subjects have a fundamental right to the protection of their data as specified in Article 1(2) of the GDPR, Article 16 of the Treaty on the Functioning of the European Union and Article 8(1) of the Charter of Fundamental Rights of the European Union. Accordingly, all data subjects must provide informed consent for all uses of their personal data (Art. 7 GDPR – Conditions for consent | General Data Protection Regulation (GDPR) [Internet] 2018). Personal data is defined as:

'personal data' means any information relating to an identified or identifiable natural person ('data subject'); an identifiable natural person is one who can be identified, directly or indirectly, in particular by reference to an identifier such as a name, an identification number, location data, an online identifier or to one or more factors specific to the physical, physiological, genetic, mental, economic, cultural or social identity of that natural person' (Art. 4(1) GDPR – Conditions for consent | General Data Protection Regulation (GDPR) [Internet] 2018)

The definition of personal data has been interpreted broadly by the Court of Justice of the European Union (e.g. Case C-434/16 *Novak*), but the GDPR only applies to living persons. Furthermore, data that has been pseudonymised remains as personal data under the GDPR. According to Article 4(5):

'pseudonymisation' means the processing of personal data in such a manner that the personal data can no longer be attributed to a specific data subject without the use of additional information, provided that such additional information is kept separately and is subject to technical and organisational measures to ensure that the personal data are not attributed to an identified or identifiable natural person (Art. 4(5)GDPR – Conditions for consent | General Data Protection Regulation (GDPR) [Internet] 2018)'

This means that only data that is truly unidentifiable becomes something that can be used beyond that to which the participant consented. In other words, aggregated data which *cannot* be linked back to the data subject falls outside of the ‘explicit’ consent category and can therefore be used for other purposes. However, this has proved problematic in relation to collection of data relating to people’s genomic information. For example, in the case of the 23andMe project, a company was providing low cost/free genetic testing, resulted in problematic use of data following the collection of sensitive lifestyle and health data, and saliva samples (Cheung 2018). It emerged that some patients believed that they were contributing their personal and genetic information for the development of treatments in the related areas. Yet, it later emerged that the company filed several patent applications and were sharing aggregated data with third parties (Cheung 2018). Whilst it was later established that 23andMe’s actions were technically lawful, their actions have been described as dishonest and immoral (Cheung 2018). This case highlights issues surrounding the appropriation of rights to share or sell on that data to other entities, but also the potential problem of aggregate data being used for commercial gain given that aggregate and unidentifiable data is outside of the explicit informed consent category of the GDPR and thus the informed consent of the data subjects would not be required. Of course, this raises ethical considerations for researchers who do not conduct research for commercial gain and receive informed consent upon this information, but where later that data is reused in an unidentified and aggregate format for commercial gain.

### Citizen science - an ethical dilemma

The importance of citizen science in terms of knowledge sharing and knowledge building has been stressed by the European Commission under the H2020 funding calls whereby such an approach to scientific research supports the E.U. Open Science and Open Access agenda (Suman and Pierce 2018; Science with and for Society - Horizon 2020 - European Commission [Internet] 2019). Nonetheless, questions about the compatibility of this agenda with the GDPR and traditional privacy ethics have come under scrutiny over the past number of years (Suman and Pierce 2018; Cheung 2018).

For example, it has been argued that data re-use is a core aspect of the Open Science agenda and yet this contradicts the ‘purpose limitation’ under the GDPR, albeit that Article 5(1)(b) states that ‘further processing for archiving purposes in the public interest, scientific or historical research purposes or statistical purposes shall, in accordance with Article 89(1), not be considered to be incompatible with the initial purposes’ (Cheung 2018; Art. 5(1)(b) GDPR – Conditions for consent | General Data Protection Regulation (GDPR) [Internet] 2018). What is important to recognise here is that complying with the latter in an ethical manner requires in-depth consideration by researchers. For example, as researchers we typically seek informed consent for the use of data for a research project and may seek consent to re-use the data for future research in a similar area, using the data in a similar manner. However, what about when the re-use is for commercial benefit? In this respect, it is argued in this paper that the re-use of data should only occur when informed consent is sought again, providing full information on the reuse intention. This would give the data subject the opportunity to change their mind and revoke their consent in a manner identical to the way that consent was obtained originally (Art. 7 GDPR – Conditions for consent | General Data Protection Regulation (GDPR) [Internet] 2018). Or at the very minimum, the data subject should be informed, at the first seeking of informed consent, that the research data may be re-used in the future and this may lead to commercial benefit of a person and/or company. Of course, it must be recognised that there is a difference here between research data being used for commercial gain and research findings that are publicly available being used for commercial gain, the latter being completely outside of the control of the research teams.

How is this different than before? Data has been described as the new oil, and its commercial value has altered significantly over the past few decades (Economist, The 2017). As such, the role of the researcher has changed because the researcher is now the collector and controller of this valuable asset and must adapt their ethical position and practices to meet these changes. For example, the authors of this paper are currently conducting research in the area of Huntington’s disease. As part of this project, we will be collecting data on the clinical care pathways and the lived experience of patients and their families. This type of data is very useful for the future development of care and management of Huntington’s disease and as researchers we are keen to advance knowledge and improve outcomes for patients with this disease. However, advancing knowledge and improving outcomes beyond the research –– through the scaling up of, for example, Connected Health interventions that are shown to improve outcomes for Huntington’s disease patients –– costs money and has the potential to make money. Therefore, we are in a dilemma because we are seeking informed consent to collect data to advance knowledge and improve outcomes for the sake of advancing knowledge and improving patient reported outcomes. In other words, we will not personally benefit, and we have outlined this in our participant information leaflet. Nonetheless, our data may be reused by a company, who in turn, may profit from the findings, and this is likely the case considering the Research and Innovation Staff Exchange (RISE), part of the EU’s Marie Skłodowska-Curie Actions. For that reason, we have outlined the potential for this to happen in our participant information leaflet to ensure that those who agree to participate are fully informed that a third party may potentially benefit from the data subjects’ participation in the study.

This is an interesting consideration because it is, on the face of it, an increased use of citizen science methods, which is honourable and inclusive, but it may throw up problems whereby it may, in fact, be exploitative. Why should someone make financial gain from others’ data whilst those providing the data do not profit from providing their data? The participants in healthcare studies are often experiencing ill-health, their very reason for being part of the study sample, unless of course they are a healthy control group. Thus, they are already vulnerable and are then providing their data to others who use them to advance knowledge and improve outcomes but also to profit. This dilemma is not an easy one to address because the data is needed to advance the state-of-the-art but there is the associated risk of potentially exploiting already vulnerable study participants. The utilitarian argument attached to informed consent, as espoused in the literature, may go some way to addressing this dilemma. This argument suggests that the minority –– the data subjects in this project whose privacy is being protected through anonymity and aggregation of data –– should not have the right to provide informed consent to the further use of the data because this data may be potentially useful for the majority (Tännsjö 2014). This is an ethical argument with strong philosophical undertones – do we protect the right of the data subject at all stages, i.e. ensure to collect their explicit consent for all re-use of the data. Or do we, decide to follow the principle that because the data has been anonymised and aggregated that it is no longer owned by the data subject and can thus be used for the greater good without their explicit consent? What about when profit will be made from this data? It is beyond this article to provide a concrete and final answer to this ethical dilemma, but rather what we have done as a research team is consider the potential for ‘function creep’, and attempted to address it within our project.

### Problem solving these issues

A review of the Irish Data Protection Act 2018 (Section 36(2)) (Health Research) Regulations 2018 seems to suggest that this issue has been somewhat dealt with in Ireland, although it has yet to be interpreted by the Courts. The 2018 Regulation states that explicit consent is required for the processing of health data. Moreover, it stipulates that the subject must be aware of the specified research to which the data will be used, in relation to a particular area, or more generally in that area, or a related area or part thereof (S.I. No. 314/2018 - Data Protection Act 2018 (Section 36(2)(c)(viii)(e)) (Health Research) Regulations 2018 [Internet 2018). This significantly limits the re-use of data in the Irish jurisdiction unless explicit consent has been provided. This suggests that all personal health data use and re-use must result from explicit consent related to a specific project in a particular area or something general to that area. Some commentators have argued that this will impact future research in the Irish healthcare setting, raising potential practical difficulties about the re-consent process and indeed the time this process might take (Clarke et al. 2019). While these are valid concerns, the push to secure explicit consent may be required in contemporary healthcare research as a result of the new value placed on personal and sensitive data. This may be a new ethical step that us as researchers are required to develop and adopt.

Gaining consent for re-use at the outset of a project has proved difficult for researchers due to the fact that there is often a lack of knowledge of how data will be re-used at the initial consent stage of a project, and therefore scholars have developed new and novel means of renegotiating forms of consent, some of which will be discussed next -.

*Open consent*, or as it is also known ‘radical honesty’ around consent, expressly excludes privacy of the data. An example of a high-profile project that adopted this approach is the Personal Genome Project run at Harvard University since 2005. Part of the informed consent process was that participants were informed that they could be identified as part of the project and could face certain risks, such as loss of insurance. This is an interesting method of gaining consent for such data collection but the risks to the participants are very high. Some scholars have described open consent as ‘ethically vague and questionable’ (Cheung 2018). *Broad consent* takes an incremental approach to collecting informed consent. Under this model the participant gives consent to a framework of future use of the data and each additional use is subject to the approval of an ethics committee to ensure that it fits within the framework (Fisher and Layman 2018). An example of a study adopting this approach is the Norwegian Mother Child Cohort Study. While this approach seems less risky than the open consent process, it has been critiqued due to the lack of participation of the study participants in the data management and use process – they tend to have a passive role under such a model (Cheung 2018). *Dynamic consent* may be the most participant friendly, in terms of respecting the original consent doctrine outlined above (‘individual autonomy, dignity, and integrity, rooted in the fundamental respect for a person, and intertwined with the right to respect privacy’ (Cheung 2018). Dynamic consent operates by providing the participants with ownership of their data through a digital platform. Through this platform, the participant can alter consent choices, engage/withdraw from new research and new uses of the data and so on. An example of the use of this type of consent process can be found in the EnCoRe project of three biobanks in Oxford from 2008 to 2012. While this form of consent appears appealing from an ethical standpoint, some important issues have been raised. For example, some have suggested that this form of consent is useful for those who are digitally literate, engaged with the process, and invested in the eHealth arena, but problematic for those outside of this category (Steinsbekk et al. 2013). An additional concern is that, unlike broad consent, there is no additional ethics committee review required for each new use of the data (Cheung 2018). Finally, dynamic consent does not take account of fluctuating capacity, which is often a reality in medical research-fluctuating capacity could be incorporated at design phase. *Portable legal consent* is a concept which allows the participant to own and control their data and provide it to whoever they wish. This model was first proposed by John Wilbanks of Sage Bionetworks who suggested that the five categories of data are genetic sequence, clinical information, medical record, patient reported outcomes, and personal sensor data (Cheung 2018; His goal was to create an open, massive, mine-able database of data about health and genomics [Internet] 2019). The portability is usually achieved through an online platform whereby an organisation can seek participants and participants can seek organisations. At all times, the participant is the controller of the data and can decide with whom they wish to share their data. This data usage usually has certain agreements attached. Broadly speaking, these include a commitment that the data will not be used to do any harm and that it will be made available in open access platforms. *Meta consent* relates to a participatory consent process whereby the participant engages in a process of deciding how and when they will choose to provide consent, at a meta level rather than a micro or granular level (Cheung 2018). What is meant by this is that the participant decides what type of consent is required for the future data use (Ploug and Holm 2015). It is unlikely that this form of consent is GDPR compliant due to its weakness on explicit consent for each use of the personal data (at a granular level).

### A case study on point

**Fig. 1:**
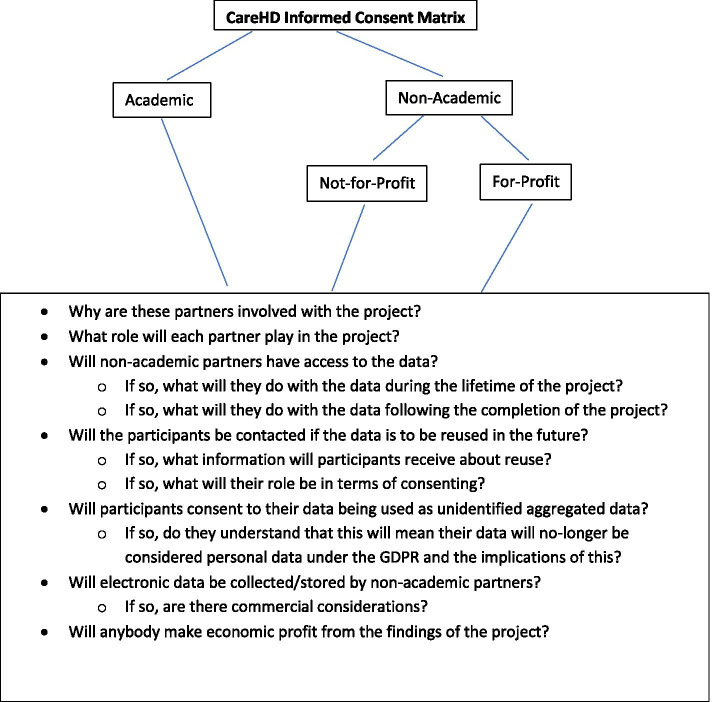
CareHD Informed Consent Matrix

This section will discuss the CareHD^1^ Informed Consent Matrix (see Fig. 1) adopted by the CareHD research project. The Matrix was designed using the current state-of-the-art and following a comprehensive ethical and data protection evaluation of the project. Having considered the various types of consent outlined above it was decided that none fully covered the requirements of the project. Therefore, the project designed a fit for purpose informed consent approach that incorporated many aspects of the above types, but went further by adding additional aspects such as consent around aggregation and uses after aggregation.

As outlined above, CareHD is a research project exploring the role of Connected Health supports in relation to community care for Huntington’s disease patients. The adoption of Connected Health in this case has the potential to support individuals living with Huntington’s disease in terms of remaining in the community and living independently for longer. A core aspect of the project is to gather data on the care pathway and the lived experience of those with Huntington’s disease. This requires an intensive engagement with both healthcare professionals and those living with Huntington’s disease. The project is a collaboration between academics, not-for-profit organisations, and for-profit organisations. As such, the project has competing agendas – academic partners are conducting research to advance the state-of-the-art; not-for-profit organisations are involved in the research to advance treatments and supports for their patients/members; and for-profit organisations are involved to advance their expertise and knowledge base, which has potential commercial benefits. As outlined above, the ethical position for the academic project partners can be understood within the traditional parameters of ethical considerations for research. However, the partnering with non-academic organisations brings an additional layer of ethical considerations. The primary identified consideration was informed consent. To ensure that the participants were fully informed it was necessary to construct a project specific informed consent matrix. In designing the project specific informed consent matrix the research team considered a number of important aspects of the project and the current state-of-the-art in the area of consent. It was concluded that the traditional forms of consent, as outlined above, could be expanded to meet the particular needs that arose during this project, namely data reuse (aggregate and non-aggregate) and the potential for commercialization. Whilst it is accepted that broad consent comes close to addressing the issues identified during this project, namely commercialization, it does not explicitly deal with the issue of the use of aggregate data sufficiently. It was considered that these issues were transferrable beyond this project and therefore applicable to most contemporary research projects, particularly those that collaborate with non-academic partners and/or intend to (or have the potential to in the future) reuse data, whether aggregated or not.

The CareHD research team decided that the data subject would be asked to agree/disagree to data re-use, agree/disagree to data re-use that might lead to commercial gain for a third party, and agree/disagree to data re-use with industry. Further, we decided that we would get explicit consent on whether the data subject wished to give explicit consent to any re-use. In addition to addressing the ethical considerations discussed above, this ensures that the project is GDPR compliant because participants have been asked for specific consent for each of those different acts of processing.

Furthermore, we adopted a similar position in terms of the data becoming ‘non-personal’ data and thus falling outside of the GDPR safety net. We decided that informed consent should still apply even when data provided becomes non-personal data, de-identified with no prospect of becoming re-identified. In an age where data is a valuable commodity, this has never been more important. The reason we have taken this position is similar to the argument put forward above. When collecting personal data under the GDPR we are restricted in what we can do with the data and have strict protocols in terms of how we comply with processing principles set in Article 5 of the GDPR (e.g. data governance, data minimisation, storage limitation etc.). Yet, these restrictions and protocols fall away, formally, when the data is no-longer deemed personal data under 4(1). Therefore, we are at liberty to reuse, share, retain etc. However, we are again confronted with a dilemma – is it to the benefit of the area under study, such as Huntington’s disease, to reuse, share, retain etc. the data as this allows for further research to be conducted in a more efficient manner because there is less ‘re-inventing the wheel’?

That said, the question must be asked, who is going to commercially benefit from the use of this data? True, the Huntington’s disease community(s) will benefit in terms of advancements in the space, but again should the re-usage of this data be done without the explicit consent of the data subject? We would argue that it should not. Having explicit consent as to re-use, even when it has become non-personal data, gives power to the data subject in terms of the future use of their data. Of course, this is particularly important when aggregating unidentifiable non-personal data, due to this data not being capable of being separated out at a later date to remove one particular data subject’s data. Thus, gathering informed consent on using a data subject’s non-personal data in an aggregated manner at the outset of a study allows for the inclusion of those who provide consent and the exclusion of those who do not. Without this, an entire data set might become unusable because the data belonging to subjects who did not consent has been integrated with the data of those who did consent, and the researcher may not be able to separate and remove the data of the former. Due to this difficulty, many do not seek consent because this data is not personal data any longer. This challenge can be overcome by seeking consent at the outset of the project for the data re-use even when it is non-personal data and may be commercially valuable to a third party. In other words, there is an onus on the researcher to gain informed consent to aggregate the data thereby outlining the potential impact in terms of reuse – outlining that the researcher may not know how the data will be reused in the future and allowing the data subject to make a decision on whether they wish their data to be used in such a manner in the future.

Other concerns arise from the use of aggregate data. For example, big data, to date, has been used successfully for understanding diseases and assisting with clinical decisions, and this must be seen as a positive in terms of population health. Yet, when health data can define a population, as opposed to an individual, this can still have significant and detrimental implications for individuals within that population. Genetic discrimination involves the “denial of rights, privileges, or opportunities or other adverse treatment based solely on genetic information (including family history)” (Erwin et al. 2010). A number of studies have highlighted the discrimination faced by Huntington’s disease sufferers and their family members as a result of the condition being inherited. For example, a study in Canada found that 40% of participants who were at high-risk of Huntington’s disease faced genetic discrimination (Bombard et al. 2009). Another potential problem is the stigmatisation of groups resulting from findings of aggregated data (Cheung 2018). Aggregated and de-identified data can be shared more easily with industry, for example, where it potentially moves from enquiry research to commercial research. The reported link between the warrior gene and aggression in the Maori community in New Zealand has also been alluded to (Cheung 2018). It has been suggested that this has wider implications than health which could span across the criminal justice system, influencing police officers and juror’s impression of those from the Maori community (Cheung 2018). These issues are important issues for researchers to consider when making final decisions on consent. Should participants be informed of the potential risks related to their data even when aggregated? This risk does not relate to them personally but to the community of people to whom the study relates, Huntington**’**s disease patients for example. It is suggested that, to be truly transparent and to seek fully informed consent, this information should be provided to the participant before they consent to participate. Indeed, scholars working in the area of ethics have suggested the adoption of a risk assessment at regular stages of the data use and re-use rather than simply at the data collection, de-identification and disclosure stage (Cheung 2018; Hon et al. 2011; Ohm 2009).

### Concluding remarks

This paper has highlighted the importance of reframing informed consent in an era of citizen science, technology and industry involvement with research, particularly in the healthcare arena. If we are truly concerned with ethics and data subjects’ rights to autonomy, dignity and privacy we must be fully transparent in terms of data generation, analysis, validation and re-use, even if this means that the researcher/research team are required to take additional and sometimes onerous steps to fully inform the data subject, even when it is not required by regulation. This full information might be that we as researchers do not currently know how their aggregate data will be used in the future, because we cannot anticipate what research it might be required for in the future, thus allowing the data subject to decide whether this is acceptable to them or not. Citizen science is about more than including the data subject as a stakeholder in the project design, implementation, and dissemination process. It is, and should be, about empowering data subjects and true collaboration between the researcher and the data subject. For this to truly occur, the data subject must be an equal stakeholder in the process and be fully informed of all elements of the process, including such issues as data re-use, even when this re-use pertains to when their data becomes non-personal data as defined under the GDPR. Without this transparency, some might argue that we are moving into the realm of covertness and that the concept of trust that permeates the citizen science model is simply a fallacy.

## Data Availability

Not Applicable.

## Footnotes

1 CareHD is a Horizon 2020 research project that aims to design patient-centred Connected Health enabled care pathways for people living with Huntington’s disease http://www.carehd.eu/
